# A Fast Anti-Jamming Algorithm Based on Imitation Learning for WSN

**DOI:** 10.3390/s23229240

**Published:** 2023-11-17

**Authors:** Wenhao Zhou, Zhanyang Zhou, Yingtao Niu, Quan Zhou, Huihui Ding

**Affiliations:** 1 School of Electronic Information Engineering, Nanjing University of Information Science & Technology, Nanjing 210044, China; 18168761685@163.com (W.Z.); dinghuihui96@163.com (H.D.); 2 The Sixty-Third Research Institute, National University of Defense Technology, Nanjing 210007, China; zzy285518423@hotmail.com; 3 College of Communications Engineering, Army Engineering University of People’s Liberation Army, Nanjing 210042, China; zq_fnn@foxmail.com

**Keywords:** imitation learning, anti-jamming communication, wireless sensor network

## Abstract

Wireless sensor networks (WSNs), integral components underpinning the infrastructure of the internet of things (IoT), confront escalating threats originating from attempts at malicious jamming. Nevertheless, the limited nature of the hardware resources in distributed, low-cost WSNs, such as those for computing power and storage, poses a challenge when implementing complex and intelligent anti-jamming algorithms like deep reinforcement learning (DRL). Hence, in this paper a rapid anti-jamming method is proposed based on imitation learning in order to address this issue. First, on-network nodes obtain expert anti-jamming trajectories using heuristic algorithms, taking historical experiences into account. Second, an RNN neural network that can be used for anti-jamming decision making is trained by mimicking these expert trajectories. Finally, the late-access network nodes receive anti-jamming network parameters from the existing nodes, allowing them to obtain a policy network directly applicable to anti-jamming decision making and thus avoiding redundant learning. Experimental results demonstrate that, compared with traditional Q-learning and random frequency-hopping (RFH) algorithms, the imitation learning-based algorithm empowers late-access network nodes to swiftly acquire anti-jamming strategies that perform on par with expert strategies.

## 1. Introduction

Over the past two decades, bolstered by the widespread adoption of the internet of things (IoT), wireless sensor network (WSN) technology has progressively evolved into a dependable, efficient, and scalable network infrastructure that caters to a plethora of IoT applications [1]. These applications include environmental monitoring [2], accurate positioning [3], and target tracking [4]. Nevertheless, due to the open nature of the wireless channels, the reliability and efficiency of communication between wireless sensor nodes are vulnerable to various types of intentional malicious jamming. One of the mainstream techniques in modern communication for anti-jamming is spread spectrum (SS) technology, which has been utilized for anti-jamming purposes since the 1950s and is still extensively employed in various wireless and mobile communication systems [5]. Though spread spectrum technology and its several modified versions have demonstrated effectiveness in countering typical types of jamming, including single-tone, multi-tone, and partial-band jamming, their capacity to adapt to a wide range of jamming settings is constrained. Consequently, individuals may encounter difficulties when seeking to adapt to a diverse array of dynamic, exceptionally effective, and novel forms of jamming.

In recent years, the advancement of machine learning has provided new research perspectives for communication anti-jamming methods, garnering widespread attention across various fields, including those associated with wireless sensor networks [6].

In contrast with supervised and unsupervised learning approaches, reinforcement learning does not necessitate the utilization of pre-existing datasets for training purposes [7]. The learning process is distinguished by the independent discovery of optimal options [8,9]. In the domain of communication, specifically pertaining to anti-jamming techniques in wireless sensor networks, it is important to acknowledge that the external jamming environment has the potential to undergo rapid fluctuations. The phenomenon of malicious jamming exhibits dynamic characteristics, with the specific type often remaining unknown. Furthermore, the intelligence displayed by such jamming further complicates the task of creating a pre-existing training dataset. In the presence of an unfamiliar jamming environment, the utilization of reinforcement learning enables the acquisition of jamming patterns in real-time, hence facilitating the steady enhancement of the transmission policy. This attribute provides a significant advantage when achieving reliable communication within complex and constantly evolving jamming environments. Consequently, to address the challenge of decision making in the face of unknown jamming techniques, relevant scholars have explored the use of model-free reinforcement learning (RL) algorithms, such as Q-learning, for anti-interference decision-making [10,11]. In addressing the challenge of anti-jamming communication for unmanned aerial vehicle (UAV) swarms, the authors of [10] proposed an intelligent and quick frequency-hopping algorithm. The algorithm aims to optimize the information transmission rate while minimizing the frequency-hopping overhead. Based on traditional Q-learning, a myopic value of perfect information is used to select the transmission channel. Subsequently, environment observation information is used to amend the Q-value in real time based on the Gauss–Gamma distribution model, so that the UAV swarm network is able to achieve anti-jamming communication with better transmission rate and frequency-hopping overhead performance under limited training overhead. Addressing the aeronautic swarm network composed of various unmanned aerial vehicles (UAVs) equipped with simultaneous transmission and reception anti-jamming radios, a multi-slot jamming sensing method is proposed in [11] based on an improved energy detection mechanism, and anti-jamming strategies based on stateless Q learning and fairness Q learning are designed, respectively, to allocate the best transmit power and frequency channel for the radio station. Simulation results verify that the stateless Q-learning strategy, which offers the highest capacity, is well suited for application scenarios that seek to maximize the capacity of individual radios. Alternatively, the fairness Q-learning-based anti-interference strategy achieves a compelling balance between network capacity and fairness, making it appropriate for scenarios in which the aim is to maximize total network capacity.

Furthermore, a novel information transmission routing strategy, founded on Q-learning principles, is introduced in [12]. This strategy facilitates the adaptive selection of suitable neighboring sensors by wireless sensors within a distributed framework, thereby promoting efficient and energy-conserving information exchange. In employing this method, wireless sensors initially acquire status and action data from adjacent sensors and utilize the compiled information to update the Q-values of neighboring sensors. Consequently, wireless sensors are able to discern optimal adjacent sensors for information transmission by considering the corresponding Q-values. The authors of [13] proposed an innovative distributed stochastic routing approach employing a mobile sink anchored in a double Q-learning algorithm, aimed at bolstering network performance within wireless sensor networks characterized by unpredictable communication links. To balance the energy dissipation between nodes, a rotation of the selected cluster head nodes is implemented, contingent upon a newly proposed threshold energy value. The simulation results demonstrate that the proposed algorithms significantly promote the learning rate, thereby diminishing data collection latency.

However, when the problem model is applied to a large state–action space, the “curse of dimensionality” problem faced by reinforcement learning leads to significantly increased convergence time and even difficulty in convergence [14], which severely affects the feasibility of such algorithms in the time-varying environments of wireless sensor networks. To address the slow convergence issue of classical reinforcement learning in large state spaces, researchers have introduced deep neural networks into reinforcement learning to handle problems with complex state spaces in wireless sensor networks [15,16,17]. The primary benefit of deep reinforcement learning (DRL) stems from the integration of the abstracting skill of deep learning (DL) with the decision-making aptitude of RL. By using neural networks to perceive high-dimensional features, it becomes possible to achieve end-to-end output, greatly reducing the complexity of the problem. In [15], to maximize network performance, a wireless sensor network’s optimal transmission strategy, based on a deep Q-learning network (DQN) and deep deterministic policy gradient (DDPG) is proposed. The optimization of unmanned aerial vehicle scheduling and power control is jointly modeled as a Markov decision process (MDP), aiming to maximize data transmission among WSN sensor nodes under opportunistic access mode. Addressing the issue of UAV-assisted data transmission in wireless sensor networks, [16] proposed an optimal transmission strategy that combines DQN and DDPG. The objective of this method is to optimize the long-term transmission of data by simultaneously optimizing the scheduling of unmanned aerial vehicles (UAVs), the allocation of bandwidth, and the transmit power of cluster heads. Given the dynamic nature of the UAV’s location, real-time transmission tasks, and the varying condition of these clusters at different time slots, the implementation of flexible scheduling strategies and effective power control mechanisms might result in enhanced data transmission performance and the decreased energy consumption of wireless sensor network nodes. Furthermore, [17] presents a perception and communication integrated framework based on DRL. Using the optimal nodes in a network, idle channels or spectrum resources are allocated to secondary users (SUs), so as to facilitate the establishment of communication links for data transmission when the primary user (PU) channels are idle.

However, the efficacy of DRL methods is diminished as a result of the substantial computational complexity and extensive training duration necessary to develop a model with a specific level of generalization capability. Hence, given the ongoing advancements in jamming and anti-jamming technologies, it is increasingly imperative to investigate and exploit advanced methodologies and potentialities in order to augment the efficacy and sophistication of anti-jamming solutions in wireless sensor networks across diverse application scenarios. Imitation learning, as a learning method that mimics expert strategies, allows simple response by imitating behaviors, extracting useful historical experiences with which to replicate expert-like behaviors in the environment. The learning process requires just a small number of expert demonstration samples. This approach for sensor communication strategy learning has the advantage of incorporating expert experience or past anti-jamming experience into the development of expert strategies. With fewer interactions, the WSN sensor nodes learn strategies which are comparable to expert demonstration samples and exhibit similar decision-making styles as the experts. This approach also demonstrates strong feasibility in algorithmic operations. The approach proposed in [18] integrates convolutional neural networks (CNN) with imitation learning. CNN is employed to capture resource management models, while imitation learning is utilized inside the RL process to decrease the training duration required to achieve the optimal strategy. This provides a more efficient strategy optimization solution for cloud resource scheduling, surpassing various current heuristic algorithms. Ref. [19] utilizes imitation learning algorithms based on demonstration sample data from multiple expert nodes in order to solve cross-layer routing problems in multi-hop CRNs, which further reduces the training cycle and speeds up the learning process.

A fast anti-jamming algorithm based on imitation learning has been proposed for multi-agent WSNs. This algorithm aims to improve the communication performance of wireless sensor networks in the presence of hostile jamming activities. To fully exploit historical experience, a heuristic-algorithm-based expert anti-jamming trajectory generation scheme is proposed. These trajectories provide the system stronger anti-jamming. After that, the expert trajectories are used in on-network nodes as samples for imitation learning in order to train a recurrent neural network (RNN) that is capable of making anti-jamming decisions for late-access nodes. Finally, the late-access network nodes receive the anti-jamming network parameters from the on-network nodes and combine them with the states of multiple past time slots during the anti-jamming process to achieve efficient communication and anti-jamming spectrum decision-making. The simulation findings provide validation for the efficacy and convergence of the proposed algorithm in scenarios that include frequency-sweeping and comb spectrum jamming.

The present paper is structured in the following manner: Section 2 of this paper offers an introductory overview of the wireless sensor system concept and delivers a concise formulation of the problem. In Section 3, the proposed method and associated workflow are presented. Section 4 presents a comprehensive examination of the simulation outcomes. The final section of the study, Section 5, serves as a conclusion and offers potential avenues for future research.

## 2. Assumptions and System Model

### 2.1. Assumpions of the System

**Figure 1:**
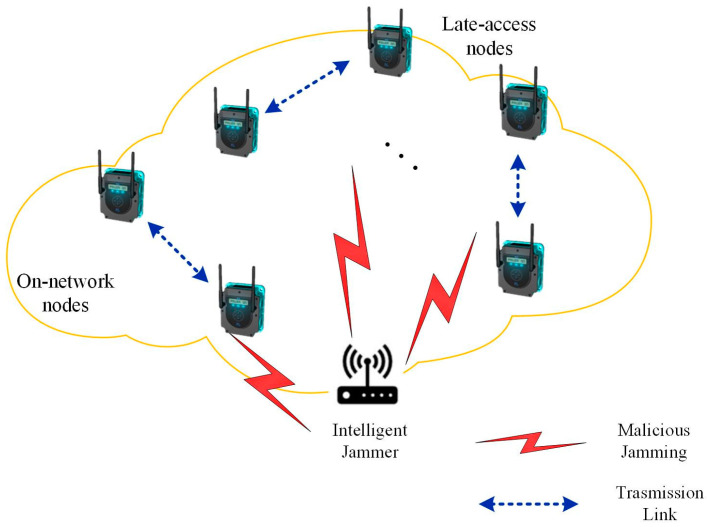
Schematic diagram of system structure.

For the sake of simplicity, the following assumptions are made for a multi-user wireless communication system to facilitate the research:

1. Considering a WSN scenario with malicious jamming sources, where the network consists of N active sensors and M available channels. The set of communication links is $N\in \left\{1,2,\ldots ,N\right\}$, with each link n∈N. The set of channels is denoted as $M=\left\{1,2,\ldots ,M\right\}$, with each channel m∈M. In this paper, each pair of active sensors is referred to as a node, which is further divided into on-network nodes and late-access nodes in the system model. An on-network node is primarily defined as a node that has been actively involved in the network for some time and has historical experience in sensing the environment and transmitting data. On the other hand, late-joining nodes are defined as nodes that join the network later and achieve fast anti-jamming communication by receiving network parameters from on-network nodes. In this paradigmatic multi-agent communication system, an unlimited number of communication transmission channels are present. The system model is depicted in Figure 1.
2. It is assumed that each agent has wideband spectrum sensing capability to detect channels where malicious jamming is present. The phenomenon of frequency switching for each transmission is denoted as an iteration, which is derived from the anti-jamming method employed by the sensor nodes.
3. It is assumed that all sensor nodes in the WSN are within the range of jamming. Malicious jamming can immediately interrupt or even intercept communication of the sensor nodes, leading to communication transmission failure. The set of interfering channels is denoted as $J=[j_{1},\cdots ,j_{K}],\;j_{k}\in M$, where k∈[1,K] represents the number of interfering channels at a given time. To ensure a stable working environment for the WSN, malicious jamming can operate continuously and consistently. Moreover, it is assumed that, by analyzing the signal waveforms of each occupied channel, jamming signals as well as legitimate signals that are received can be efficiently identified.
4. The nodes are differentiated into three modules based on their functionalities, including transmission, sensing and learning: the transmission module in the transmitter, during a uniform time slot, maintains a constant transmission power and channel; the receiver’s sensing module perceives the spectrum environment at evenly divided intervals within a time slot; the learning module in the receiver is responsible for learning the transmission strategy of the transmitter, and assigns action instructions to the transmitter at the conclusion of each time slot.
5. It is assumed that the sensor nodes in the wireless sensor network share communication time, with each time slot strictly synchronized. All sensor nodes possess identical sensing capabilities, and under identical conditions, they yield identical sensing results. Furthermore, for simplicity’s sake, it is assumed that the duration of external malicious interference is the same and is synchronized with the communication time within the sensor network.

### 2.2. System Model and Problem Formula

In this paper, the Markov decision process (MDP) is used to model wireless sensor networks. The model can be mathematically formulated as a sophisticated four-tuple $\langle S,A,P,R\rangle$, where S represents environment state space, A represents the action space of a link, P epitomizes a state transition probability function interpreting the probable distribution of impending states corresponding to a given state and each link’s action and R represents reward function.

1. State S: Wideband spectrum sensing is commonly used in sensor nodes of a WSN to obtain information about the surrounding environmental conditions. This study introduces the power spectral density function (PSD) [9] as a means to provide a more comprehensive description of the spectral environment of sensor nodes. At a given moment, the received signal power spectral density of the communication link is defined as:

(1) $$d_{t}\left(f\right)=\sum_{n=1}^{N}g_{n,t}\cdot N_{t}\left(f-f_{n,t}\right)+g_{t}^{\prime }\cdot J_{t}\left(f-f_{k,t}^{\prime }\right)+U_{t}\left(f\right)$$

In this context, the variables $g_{n,t}$ and $g_{t}^{\prime }$ are used to represent the transmission channel gain and jammer. $N_{t}\left(f\right)$ denotes the power spectral density functions of the baseband signal of the sensor node. Additionally, $J_{t}\left(f\right)$ indicates the jamming baseband signal, and $U_{t}\left(f\right)$ represents the noise. The power sample sensed at time t for the i time slot is provided as follows:

(2) $$P_{t,i}=10\log[\int_{f_{L}+(i-1)\Delta f}^{f_{L}+i\Delta f}d_{t}\left(f\right)df]$$

where, $f_{L}$ is the perceived initial frequency.

The representation of the average received power ${\bar{P}}_{m,t}$ on channel $f_{m}$ at time t can be derived from the expected signal characteristics and jammed signal parameters of the receiving node:

(3) $$\begin{array}{cc} {\bar{P}}_{m,t} & =p_{n,t}g_{n,t}\delta \left(f_{n,t}=f_{m}\right)\\ & +\sum_{n^{\prime }\in N/n}p_{n^{\prime },t}g_{n^{\prime },t}\delta \left(f_{n^{\prime },t}=f_{m}\right)+p_{k}^{\prime }g_{t}^{\prime }\delta \left(f_{k}^{\prime }=f_{m}\right)+u_{t} \end{array}$$

where $\delta \left(•\right)$ is the indicator function, that is, when x is true, $\delta \left(x\right)=1$ and when x is false, $\delta \left(x\right)=0$; $n^{\prime }$ denotes the other links except for link n; $p_{n,t}$ and $p_{k}^{\prime }$ represent the expected signal transmit power and the corresponding jamming transmit power on the link, respectively; $g_{n}$ and $g^{\prime }$ represent the transmission channel gain and the jamming channel gain, respectively; $p_{n,t}\cdot g_{n,t}\cdot \delta \left(x\right)$ and $\sum_{n^{\prime }\in N/n}p_{n^{\prime },t}\cdot g_{n^{\prime },t}\cdot \delta \left(x\right)$ are the aggregate power of all received signals on channel m, $p_{k}^{\prime }\cdot g_{t}^{\prime }\cdot \delta \left(x\right)$ is the received jamming signal, and $u_{t}$ is the noise power.

This research aims to establish the environmental condition of the T timeslot by considering the combined received power observed on the M channels within that specific timeslot:

(4) $$s_{T}=[P_{1,T},\ldots ,P_{M,T}]\in S$$

2. Action A: Because the action of each pair of sensors is chosen from a set of M available channels, the action a chosen by any node n is defined as an array of size M×1. When the m channel is chosen for communication, the m element of a is set to 1, while all other elements are set to 0:

(5) $$a_{T}=\left[{\hat{a}}_{1},\cdots ,{\hat{a}}_{m},\cdots ,{\hat{a}}_{M}\right]$$

where ${\hat{a}}_{m}$ indicates whether channel m has jamming or not, which is expressed as follows:

(6) $${\hat{a}}_{m}=\left\{\begin{array}{l} 0,j_{k}\neq m\\ 1,j_{k}=m \end{array}\right.$$

3. State transition probability P: $S\times A\times S\prime \to \left[0,1\right]$ represents the probability of the set of agents transitioning to state S after taking action A in the channel state.

4. Reward R: In the process of data transmission, the degree of jamming in the communication is determined and evaluated by the received signal-to-jamming-noise ratio $\left(R_{SJNR}\right)$ of the sensor nodes of WSN, in order to evaluate the effect of this communication, as indicated by Equation (7) below:

(7) $$\theta_{n,t}=\frac{g_{n}\cdot p_{n,t}}{\int_{f_{n,t}-\Delta B/2}^{f_{n,t}+\Delta B/2}\left[\sum_{n^{\prime }\in N/n}g_{n^{\prime }}\cdot N_{t}\left(f-f_{n^{\prime },t}\right)+g^{\prime }\cdot J_{t}\left(f-f_{k,t}^{\prime }\right)+U\left(f\right)\right]df}$$

where, ΔB indicates the channel bandwidth.

The data transmitted through the wireless sensor network communication link n needs to satisfy signal-to-jamming-noise ratio $\left(R_{SJNR}\right)$ $\theta_{n,t}\geq q_{d}$ at any arbitrary time in timeslot T, denoted as $\theta_{n,T}\geq q_{d}$. Here, $q_{d}$ is the threshold of the received signal-to-jamming-noise ratio. In this study, the normalized throughput of data transmission for the link n is defined as $c_{n,T}=\delta \left(\theta_{n,T}\geq q_{d}\right)$, and the overall normalized throughput of the wireless sensor network is defined as $c_{T}=1/N\sum_{n=1}^{N}\delta \left(\theta_{n,T}\geq q_{d}\right)$.

The reward function is formulated as a mathematical function that takes into account the state space and the current action space, and assesses the impact of the current action performed by the node in the present state. The representation of the instantaneous reward is as follows:

(8) $$r_{n}\left(s_{T},a_{T}\right)=c_{n,T}$$

The average global reward obtained by each link performing action a in the environment state $s_{T}$ is expressed as follows:

(9) $$R\left(s_{T},a_{T}\right)=1/N\sum r_{n}\left(s_{T},a_{T}\right)$$

## 3. Fast Anti-Jamming Algorithm Based on Imitation Learning Description

According to the communication anti-jamming scenarios in the aforementioned WSN, a fast anti-jamming method based on imitation learning is proposed in this paper. This method can effectively utilize the historical anti-jamming experiences of nodes in the network to form an expert policy and then apply this policy to the late-access nodes for efficient spectrum decision-making.

Imitation learning, as a machine learning paradigm, largely involves training agents by leveraging expert demonstration samples. In contrast with RL, imitation learning does not require designing a reward function, as it can be challenging to construct an appropriate reward function in certain cases. The primary benefit of imitation learning is its utilization of offline instruction with online decision-making methodology [20].

The historical experience samples of nodes in the network are generated using a heuristic-based approach [18] and are subsequently gathered as training data samples in this study. Following this, the high-quality demonstration samples are subsequently utilized to train an offline imitation learning recurrent neural network (RNN) model. The trained anti-jamming network parameters can be deployed and applied to the late-access nodes in the WSN. Effective communication transmission can be achieved by effectively simulating optimal decision-making patterns (i.e., selecting the next communication channel) during the anti-jamming process that are based on the states of multiple past time slots.

### 3.1. Signal Time–Frequency Analysis

**Figure 2:**
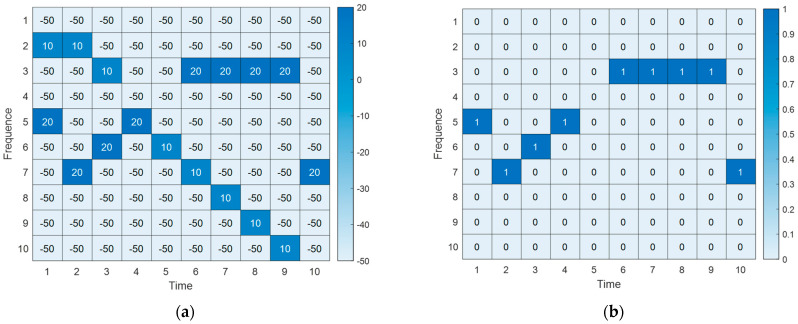
Signal time−frequency conversion analysis diagram. (**a**) Time−frequency state matrix. (**b**) Jamming time−frequency state matrix.

As mentioned above, the state $s_{T}$ defined by Formula (4) represents the signal power received by node at time slot T, including legitimate signals and jamming signals. The state $s_{T}$ of the $T_{MAX}$ time slot in the network node can be continuously recorded, resulting in a time–frequency state matrix as shown in Figure 2a, where the matrix elements represent received signal power (Unit: dBm). Here, 20 dBm represents the received jamming power and 10 dBm represents the power of the legitimate signal. Subsequently, through waveform analysis [21], the jamming time–frequency state matrix can be extracted from the time–frequency state matrix. This is shown in Figure 2b, where 1 indicates the presence of jamming and 0 indicates no jamming. The expression is as follows:

(10) $$S=\left[s_{T-T_{MAX}}^{T},s_{{}_{T-T_{MAX}+1}}^{T},\cdots ,s_{T}^{T}\right]$$

The imitation learning neural network constructed in this paper takes the jamming time–frequency state matrix S as input and outputs actions a.

### 3.2. Experience Sample Generation at On-Network Nodes

In order to train a neural network for anti-jamming decision-making, the network nodes need to generate the corresponding expert anti-jamming trajectories based on the accumulated historical jamming time–frequency state matrix using a heuristic algorithm. These expert trajectories serve as the experiential samples with which to train the neural network and represent the mapping relationship between the accumulated history S and the optimal anti-jamming actions a. The process of training the neural network using expert trajectories is known as imitation learning.

Imitation learning primarily employs supervised learning to mimic the historical experience samples (i.e., optimal anti-jamming actions) in the network nodes. Hence, the objective of this stage is for the algorithm to select trajectories based on the experienced policies of the network nodes using the jamming time–frequency state matrix S, ensuring that the total reward corresponding to the selected communication trajectory is maximized. This guarantees the selection of suitable channels for communication among the network nodes, effectively avoiding jamming.

The procedure outlined in Algorithm 1 consists of the following steps:
**Algorithm 1: Multi-agent expert trajectory generation based on heuristic algorithm****1.** **Input**: Jamming time–frequency state matrix **H** (M*T)**2.** **Output**: Expert selection sequence **Group** (N*T)Expert selection sequence initialization **Group** = zeros(*N*,*T*);**3.** **For** ii = 1:UserNumDetermine the jamming-free starting time slot on each frequency S1;  Determine the end time slot with jamming on each frequency S2;**4.** **While** min(Group(:, ii))==0 **5.**      Determine the end time slot J of continuous idle channels starting from time S1;**6.**   **For** kk = 1:M **7.**      **If** sum(**H** (S1:S2,kk)>0)==0**8.**       J(kk) = S2 + 1;**9.**      **Else****10.**      J(kk) = Find(H(S1:S2,kk) > 0.1) + S1 − 1;**11.**      **End****12.**   **End****13.**   [T,M] = max(J);**14.** Group (S1:T − 1,ii) = M*ones(M − S1,1);**15.**  S1 = T;**16.** **End**

The expert-selected action corresponding to the T time slot of node n in Algorithm 1 is:

(11) $$a_{ET}^{\left(n\right)}\left(T\right)=\left\{a_{Group\left(T,n\right)}=1\right\}={\left[{\hat{a}}_{1},\cdots ,{\hat{a}}_{m},\cdots ,{\hat{a}}_{M}\right]}^{T}$$

The expert-selected actions of the n user are concatenated to form the multi-user expert action corresponding to the T time slot, denoted as:

(12) $$a_{ET}^{\mathrm{Multi}}\left(T\right)=\left[a_{ET}^{\left(1\right)}\left(T\right),a_{ET}^{\left(2\right)}\left(T\right),\cdots ,a_{ET}^{\left(n\right)}\left(T\right)\right]$$

The expert actions from multiple time slots are merged to form an expert trajectory:

(13) $$A_{ET}=\left[a_{ET}^{\mathrm{Multi}}{\left(1\right)}^{T},a_{ET}^{\mathrm{Multi}}{\left(2\right)}^{T},\cdots ,a_{ET}^{\mathrm{Multi}}{\left(T\right)}^{T}\right]$$

### 3.3. Imitation Learning Neural Network Training

In this section, a detailed description is provided for the training of neural networks for on-network node imitation learning. In [22], imitation learning learns behavioral strategies from expert tracks to reduce exploration time.

The paper introduces a heuristic-based algorithm, which performs trajectory selection based on changes in the state prior to the given moment. The expert trajectory $A_{ET}$ obtained after training in Equation (13) satisfies:

(14) $$A_{ET}=\arg\max\sum_{T=1}^{T_{MAX}}R\left(s_{T},a_{T}\right)$$

In the actual anti-jamming process, maximum rewards are achieved by adjusting the network parameters θ and observing the historical jamming information:

(15) $$a_{T}=\underset{\theta }{\arg\max}R\left(s_{T},a_{T}\right)=\underset{\theta }{\arg\max}R\left(f_{\theta }\left(s_{T-d},s_{T-d+1},\cdots ,s_{T-1}\right),s_{T}\right)$$

By using the trained RNN neural network, the obtained $f_{\theta }\left(\cdot \right)$ is used as the decision function for the next step. Therefore, the next action $a_{T+1}$ is given by:

(16) $$a_{T+1}=f_{\theta }\left(s_{T-d},s,\cdots ,s_{T-1}\right)$$

In this paper, primary utilization is made of the input and output of the delayed-on-network node for the past d rounds in order to predict the next communication frequency. Therefore, the state input is defined as:

(17) $$S_{\mathrm{input}}=\{S_{T-d},S_{T-d+1},\cdots ,S_{T}\}$$

The action input is defined as:

(18) $$A_{\mathrm{input}}=\{A_{T-d},A_{T-d+1},\cdots ,A_{T}\}$$

The output of the next action is defined as:

(19) $$A_{T+1}=f_{\theta }\left(S_{\mathrm{input}};A_{\mathrm{input}}\right)$$

Assuming that the activation function from the input layer to the hidden layer is Z(x), then the representation of the hidden layer can be defined as:

(20) $$Y=Z(w_{f_{1}}S_{\mathrm{input}}+w_{f_{2}}A_{\mathrm{input}}+b_{0})$$

where, $w_{f_{1}}$ represents the weight parameter set for the hidden layer state input, $w_{f_{2}}$ denotes the weight parameter set for the hidden layer state input, and $b_{0}$ is the bias vector. The activation function for the hidden layer is denoted as:

(21) $$Z(x)=\frac{e^{x}-e^{-x}}{e^{x}+e^{-x}}$$

The activation function used in the hidden layer exhibits favorable mathematical properties and non-linear characteristics. It compresses the input values within the range of $\left[-1,1\right]$ and effectively handles non-linear real-world problems. This enables neural networks to adapt to complex models and data distributions, enhancing the network’s expressive power and learning capability.

**Figure 3:**
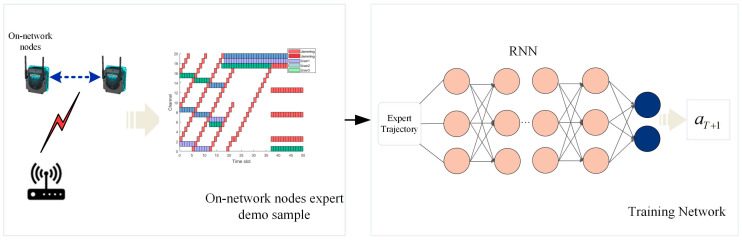
Workflow framework based on imitation learning.

Figure 3 illustrates the workflow framework of anti-jamming in wireless sensor networks based on an imitation learning algorithm.

## 4. Simulation Results and Analysis

This section presents the verification of the effectiveness of the imitation learning-based multi-agent rapid anti-jamming communication algorithm in wireless sensor networks through simulations.

### 4.1. Parameter Settings

The relevant parameters in the simulation are established according to the following configuration (Table 1):

This study considers three types of jamming: frequency-sweep jamming, comb spectrum jamming and comb frequency sweeping jamming. To effectively evaluate the performance of the proposed algorithm, this paper compares it with the following two algorithms:

✧ Q-learning algorithm: The agent constantly interacts with the environment, though only through local learning results.
✧ Random hopping frequency (RHF): All agents have the capability to randomly select the communication channel for transmission.

**Figure 4:**
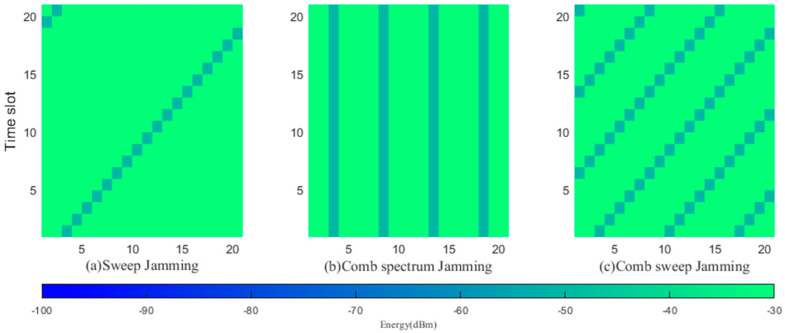
Time−frequency graphs of jamming.

For the above communication scenario, the following jamming models are adopted. Figure 4 shows the spectrograms of three types of jamming patterns: frequency-sweep jamming, comb spectrum jamming and comb frequency sweeping jamming.

This study employs a scenario wherein the jamming machinery alternates between sweep jamming, comb spectrum jamming, and comb sweep jamming. Taking T timeslots as a sample, each sample is divided into ℵ parts with long random timeslot lengths and their jamming parameters are randomly generated. There are ℵ−1 time nodes, $1<T_{1}<T_{2}<\cdots <T_{ℵ-1}<T$, such that

(22) $$\begin{array}{c} \left[s_{1}:s_{T_{1}}\right]=J_{1}\left(t\right)\\ \left[s_{T_{1}+1}:s_{T_{2}}\right]=J_{2}\left(t\right)\\ \vdots \\ \left[s_{T_{ℵ-1}+1}:s_{T}\right]=J_{ℵ}\left(t\right) \end{array}$$

here, $J_{1}\left(t\right),\cdots J_{ℵ}\left(t\right)\in \left\{J_{S}\left(t\right),J_{C}\left(t\right),J_{W}\left(t\right)\right\}$,$J_{S}\left(t\right)$ is sweep jamming, $J_{C}\left(t\right)$ is comb spectrum jamming, and $J_{W}\left(t\right)$ is comb sweep jamming.

In addition, the effect of the number of hidden layers and the sample delay on the performance of the communication network was investigated as follows.

The number of hidden layers: this mainly refers to the hidden layers added during the training of the neural network. Having more hidden layers can provide greater representational capacity, allowing the neural network to better model complex patterns and data. However, if the number of hidden layers is larger than needed, the network may overfit the training set, leading to a decrease in performance on new samples. In addition, increasing the number of hidden layers also increases the number of parameters and computational complexity of the network, resulting in longer training times and greater consumption of computational resources. If time and resources are limited, too many hidden layers may result in excessively long training times or an inability to fully train the network.

The number of sample delays: this mainly refers to the number of samples trained in past time slots. Increasing the number of past samples can typically provide more contextual information, but is not always better. The degree of correlation between past samples and current samples is an important factor to consider. If the dependency relationships in the problem are only present in the most recent past samples, using too many past samples may introduce irrelevant information and increase noise. Additionally, increasing the number of past samples increases the number of parameters and the computational complexity of the recurrent neural network (RNN). If the available amount of data is small, using too many past samples may lead to overfitting, and excessive model complexity may make model training difficult. Therefore, by using techniques such as cross-validation, different numbers of past samples can be experimentally tested on the training set and validation set, and the number that produces the best performance can be selected. The goal of such experiments is to find the optimal balance between model representational capacity and overfitting.

Among these, the scaled conjugate gradient algorithm is used in training. The advantages of this algorithm include fast convergence, high memory efficiency and good parallelism ability. It can handle multiple iterative processes at the same time and accelerate the solution speed. Additionally, in the experiments, an early termination of training is set after six consecutive increases in error on the validation set. That is, when the performance of the model on the validation set is no longer improved, the training will be terminated in advance.

### 4.2. Simulation and Analysis

**Figure 5:**
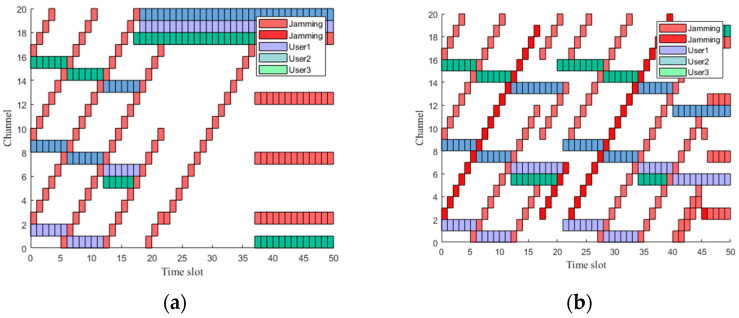
Expert demo samples generated based on heuristic algorithms. (**a**) Graphs with three types of jamming. (**b**) Graphs with two types of jamming.

According to the above parameter settings, the following analysis was performed:

Figure 5 visually illustrates the expert trajectory communication spectrogram based on the heuristic algorithm under frequency-sweeping jamming, comb spectrum jamming, and comb frequency-sweeping jamming. Specifically, it illustrates the schematic representation of the interference–resistant channel selection. In Figure 5, the green blocks represent sensor node 1, the purple blocks represent sensor node 2, the blue blocks represent sensor node 3, and the red blocks represent jamming in the channel.

Figure 5a presents the channel selection schematic under the simultaneous presence of frequency-sweeping jamming, comb spectrum jamming and comb frequency-sweeping jamming. It is evident that under three types of jamming, the sensor nodes can effectively avoid the jamming. Figure 5b shows the anti-jamming channel selection schematic under the presence of comb spectrum jamming and comb frequency sweeping jamming. During slot changes, sensor nodes 1, 2, and 3 can all effectively avoid jamming and select communication channels, demonstrating the excellent performance of the heuristic algorithm in expert trajectory generation.

**Figure 6:**
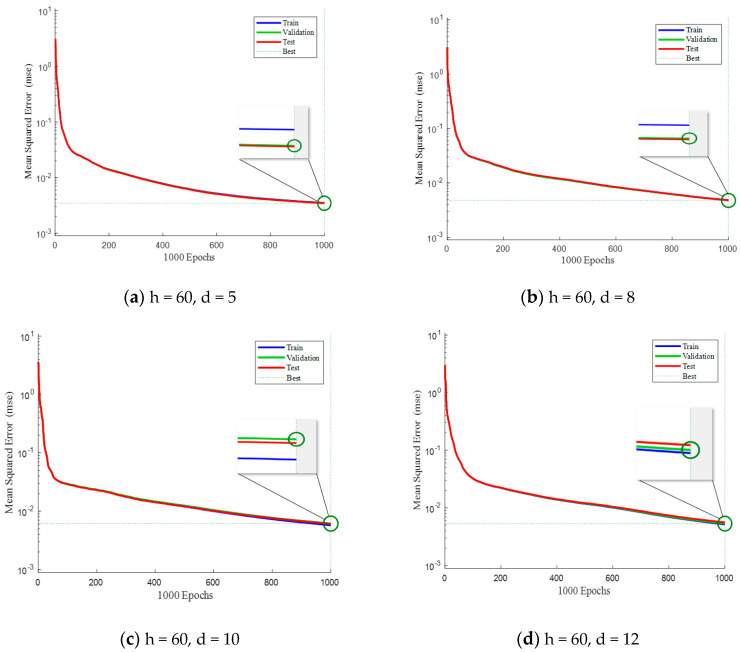
Comparison of sample training performance when the hidden layer is 60.

**Figure 7:**
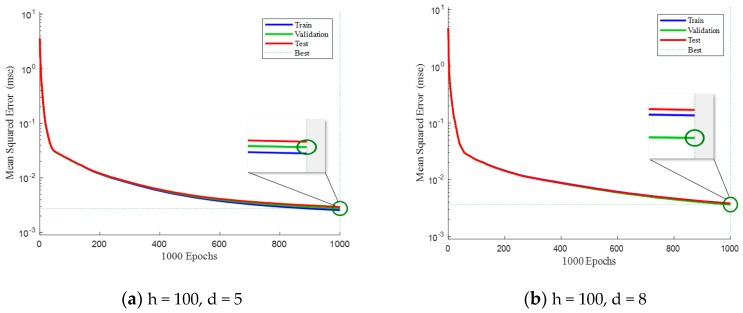

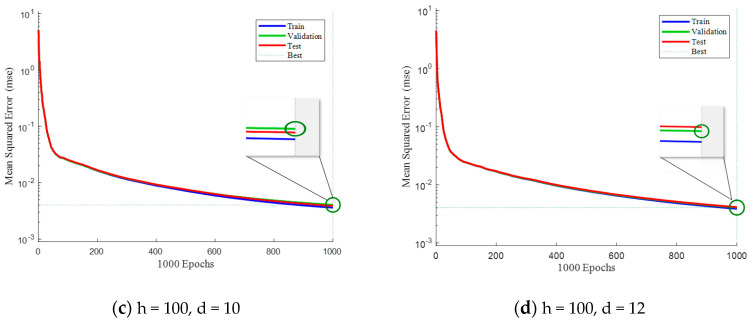
Comparison of sample training performance when the hidden layer is 100.

Figure 6 and Figure 7, shown below, mainly study the influence of different parameters on the training of neural networks. This paper mainly focuses on the number of hidden layers and the number of delays of neural networks. Under different parameters, experimental analysis is carried out to observe the advantages and disadvantages of its test performance.

Figure 6 illustrates a comparison of mean squared errors (MSE) among the training set, validation set, and testing set when the number of hidden layers is fixed at 60. The blue line represents the MSE during training, the red line represents the MSE during testing, and the green line represents the MSE during validation. By comparing Figure 6a,b, it can be observed that setting the delay as 5 or 8 has minor impact on the testing performance, as both are close to the validation baseline. However, when the delay is set to 12, the MSE in testing after 1000 iterations exceeds the MSE in validation. Therefore, it can be inferred that appropriately increasing the number of past samples normally provides more information and improves performance. However, using overly numerous past samples may introduce irrelevant information and increase noise, leading to overfitting during model training.

Figure 7 presents a comparison of mean squared errors (MSE) among the training set, validation set, and testing set when the number of hidden layers is fixed at 100. By comparing Figure 6a and Figure 7a, it can be observed that when the delay is relatively constant, a larger number of hidden layers in the neural network leads to smaller MSE in the testing set. This observation holds true and can be seen in the comparisons between Figure 6b and Figure 7b, as well as between Figure 6d and Figure 7d. Consequently, appropriately increasing the number of hidden layers can effectively reduce the MSE and improve the network’s fitting performance.

**Figure 8:**
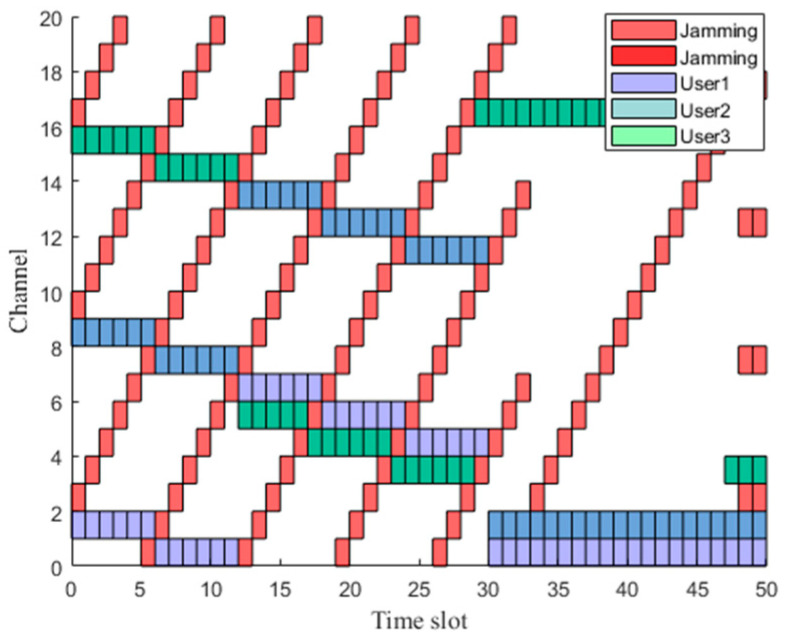
Communication time–frequency graph after training.

Figure 8 represents the spectrograms of communication applied to delayed network nodes after training. That is, the channel selection method obtained through the imitation strategy after real-time decision-making. It is apparent that the delayed network nodes can effectively avoid jamming in most time slots. Therefore, there are varying degrees of jamming in time slots 29, 47, and 48. In general, the proposed method can effectively solve the spectrum decision problem under frequency-sweep jamming, comb spectrum jamming and comb frequency-sweeping jamming.

**Figure 9:**
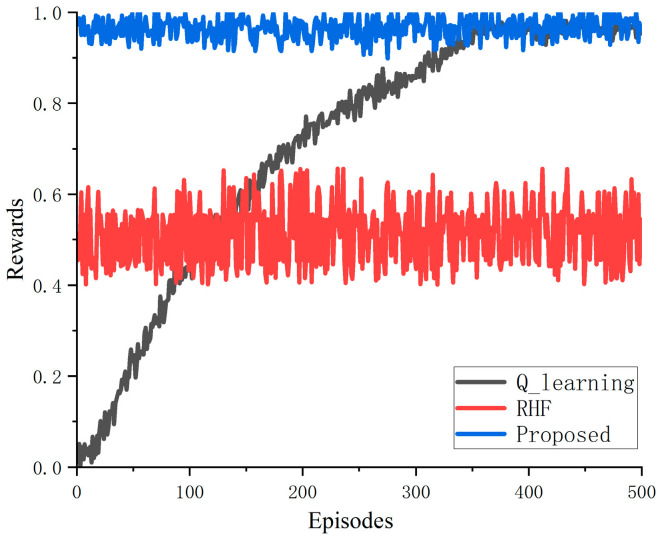
Comparison diagram of episodes–rewards under different algorithms.

As the main objective of reinforcement learning algorithms is to maximize the reward of a trajectory, a simple comparison was conducted in terms of convergence speed to understand why the algorithm proposed in this paper outperforms several alternative algorithms. As can be seen from Figure 9, the convergence value of the proposed algorithm is significantly higher than that of the communication anti-jamming algorithm based on Q learning, and much higher than that of the random frequency-hopping algorithm. By the 500th iteration, the anti-jamming algorithm based on imitation learning is stable at 0.94, the convergence value of the algorithm based on Q learning is stable at 0.9, and the convergence value of the random frequency-hopping algorithm is stable at 0.5. Therefore, the convergence value of the proposed algorithm is about 0.04 percentage points higher than that of the Q-learning algorithm and about 0.44 percentage points higher than that of the random frequency-hopping algorithm. Because the imitation learning algorithm directly utilizes the expert strategies in the demonstration samples, the proposed algorithm does not have great fluctuation in the convergence speed.

## 5. Conclusions

In this paper, an innovative imitation learning-based multi-agent intelligent communication anti-jamming algorithm is proposed for wireless sensor networks. This algorithm utilizes imitation learning to enhance the decision-making efficiency of wireless sensor networks by guiding the exploration direction of agents by imitating the decisions made by experts. Through further comparisons in terms of transmission and convergence, it can be seen that this method outperforms existing Q-learning algorithms and traditional random-hopping algorithms in terms of both transmission and convergence of wireless sensors. The convergence value of the proposed algorithm is about 0.04 percentage points higher than the Q-learning algorithm and about 0.44 percentage points higher than the random frequency-hopping algorithm. However, this method has limited generalization capability and limited ability to handle jamming patterns beyond those contained in the training samples. In future plans, there will be a continued exploration and integration of reinforcement learning algorithms, the such as DQN, AC [23], and PPO [24] algorithms, into the field of communication anti-jamming. An exploration of the merger between imitation learning, deriving insights from past data, and RL, adapting to instantaneous alterations in the present environment, is warranted. Additionally, as the proposed algorithm is still in the theoretical research stage, our team has successfully employed the Q-learning algorithm in the context of wireless communication anti-jamming projects, resulting in satisfactory outcomes. In our preliminary approach, we propose placing greater emphasis on the outcomes of RL in cases where historical samples are limited, or where historical experience has low anti-jamming reference value to the current scene. Conversely, we advocate for an increased reliance on the results of imitation learning when a sufficient quantity of state experience samples have been accumulated for its implementation. By integrating these methods, we can confer the advantages of both the flexibility of RL and the superior precision of imitation learning onto our system. Therefore, future work will also focus on hardware testing in order to validate the effectiveness of the algorithm proposed in this paper.

## Figures and Tables

**Table 1:** Settings of model-related parameters.

Description	Symbol	Value
Number of channels	M	20
Time slot length	T	50
Hidden layers	L	100
Delay number	d	10
Spectrum sensing resolution	Δf	100 kHz
Available transmission power	p	20 dBm
Jamming power	$p^{\prime }$	10 dBm
Received signal-to-noise ratio threshold	$q_{d}$	20 dBm
Maximum number of interfering channels	K	1

## Data Availability

The data presented in this study are available on request from the corresponding author. The data are not publicly available due to privacy.
